# Laparotomy enables retrograde dilatation and stent placement for malignant esophago-respiratory fistula

**DOI:** 10.1186/1477-7819-6-8

**Published:** 2008-01-26

**Authors:** Alexander Rehders, Kenko Cupisti, Marcus Schmitt, Marc A Renter, Patrick Kröpil, Özcan Iskender, Wolfram T Knoefel

**Affiliations:** 1 Klinik für Allgemein-, Viszeral- und Kinderchirurgie, Heinrich Heine Universität, Düsseldorf, Germany; 2 Klinik für Gastroenterologie, Hepatologie und Infektiologie, Heinrich Heine Universität, Düsseldorf, Germany; 3 Institut für diagnostische Radiologie, Heinrich Heine Universität, Düsseldorf, Germany

## Abstract

**Background:**

Malignant esophageal stenosis with complete obstruction and esophagorespiratory fistula (ERF) is difficult to treat with standard endoscopic techniques.

**Case presentation:**

We report a patient in whom with local recurrence of esophageal carcinoma an esophagotracheal fistula occurred. Initially the patient had undergone esophageal resection with interposition of a gastric tube. Due to complete obstruction of the lumen by recurrent tumor conventional transoral stent placement failed. For retrograde dilatation a laparotomy was performed. Via a duodenal incision endoscopic access to the gastric tube was achieved. Using a guidewire the esophageal obstruction was traversed and dilated. Then it was possible to place an esophageal stent via an antegrade approach.

**Conclusion:**

Open surgery enables a safe access for retrograde endoscopic therapy in patients who had undergone esophageal resection with gastric interposition.

## Background

Esophageal cancer is an aggressive tumor with unfavorable prognosis. Despite the radical surgery, local recurrence occurs in up to 21% of the cases [1]. Dysphagias as well as esophago-respiratory fistulae (ERF) are predominant symptoms of local tumor recurrence and represent devastating and life threatening complications. Patients are often unable to swallow food or even their own saliva without aspiration. Unless sufficient palliation is instituted rapidly, the usual cause of death is pulmonary sepsis resulting from chronic aspiration. Since covered and self expandable stents have been introduced, successful palliation has been reported in most patients[2,3]. The endoscopic management of malignant obstruction and ERF is technically challenging and requires careful endoscopic dilatation with wire guided dilators. Despite of sophisticated endoscopic strategies in some patients the passage of a guide wire is technically impossible due to a completely obstructed lumen. In this situation retrograde endoscopic dilatation via a radio guided percutaneous gastrostomy is a second option[4].

However in patients who underwent esophageal resection and transformation of the stomach into a small gastric tube for esophageal reconstruction, retrograde access is far more challenging, since not even radio guided procedures seem applicable. To our knowledge a suitable therapeutic approach in this difficult palliative situation has not been described before. We recently encountered a patient with local recurrence after esophageal resection and interposition of a gastric tube. Due to complete obstruction and ERF, he required a laparotomy for retrograde passage and subsequent stent placement.

## Case presentation

**Figure 1:**
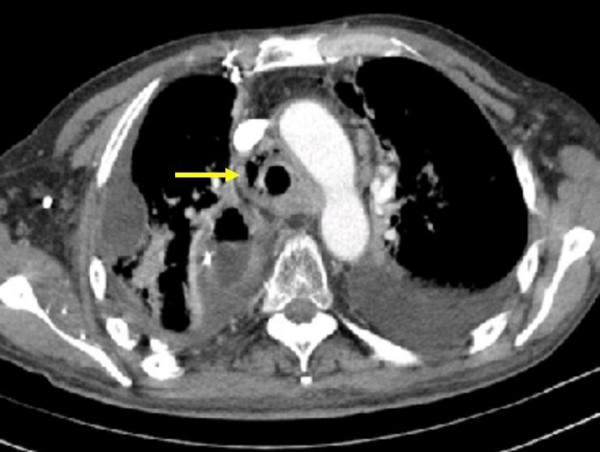
Axial contrast enhanced CT image showed a mediastinal tumor with mediastinal air and a perforation of the tracheal wall (arrow).

**Figure 2:**
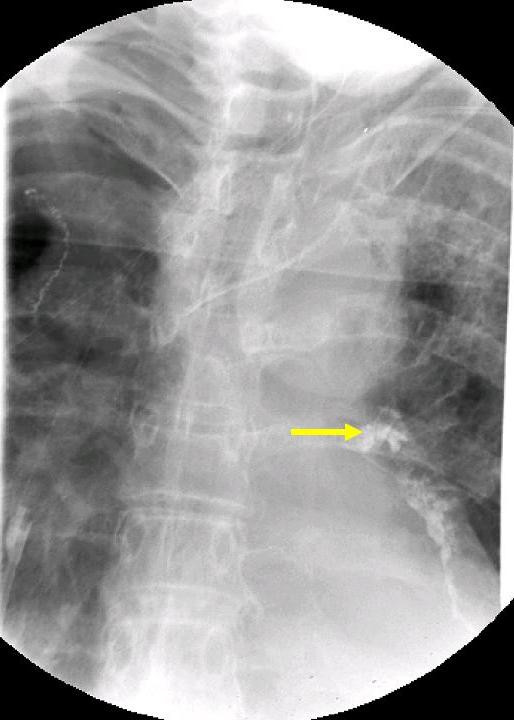
Gastrographin contrast swallow showed that oral contrast medium has entered the left sided tracheobronchial system (arrow).

A 66-year-old man presented with severe dysphagia, weight loss and recurrent pulmonary infections due to an esophago-tracheal fistula. Due to squamous cell carcinoma of the esophagus 17 month ago, he had undergone esophageal resection and interposition of a gastric tube with cervical anastomosis. This treatment was followed by adjuvant radio-chemotherapy (50 Gy with 5-FU and Cisplatin). The first signs of dysphagia developed 8 weeks before admission to our hospital. Initial endoscopic therapy revealed local tumor recurrence beginning at 21 cm from front incisors, but failed to provide palliation of dysphagia. The distal end of the stenosis could not be measured precisely due to high grade stenosis which could not be passed endoscopically. Though intravenous hyperalimentation was administered, the patient kept on losing weight. Furthermore recurrent pulmonary infections occurred and swallowing of salvia, without coughing became impossible. For palliative surgical treatment the patient was transferred to our institution. Unfortunately we found the esophageal lumen to be completely obstructed by recurrent tumor. Moreover the tumor had invaded the trachea and had caused an esophago-tracheal fistula. The fistula itself could not be seen endoscopically, but was found by gastrographin swallow and CT-scan (Figure 1, Figure 2). According to CT-scan we estimated it to be located at about 2–3 cm distal from the beginning of the stenosis. The recurrent mediastinal tumor was estimated to have a length of 6 cm and infiltrated the gastric tube.

**Figure 3:**
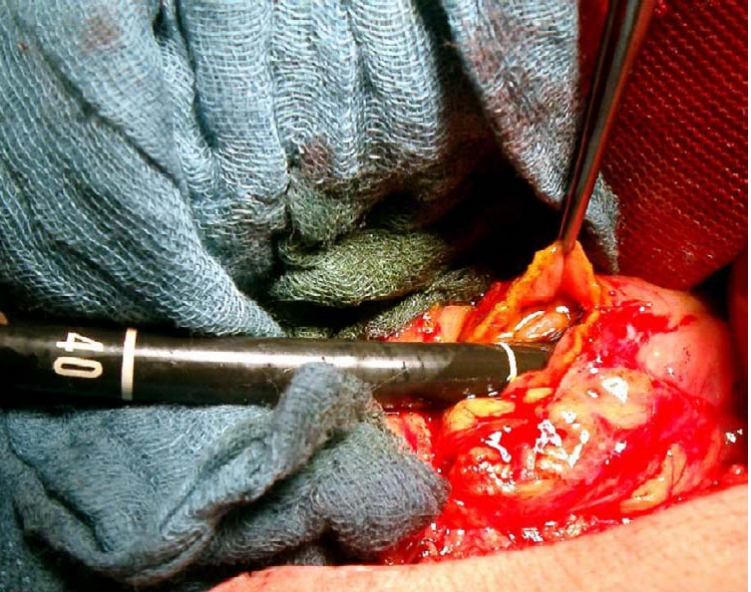
Retrograde endoscopic access to the esophageal lumen was obtained by open surgery and a duodenotomy.

**Figure 4:**
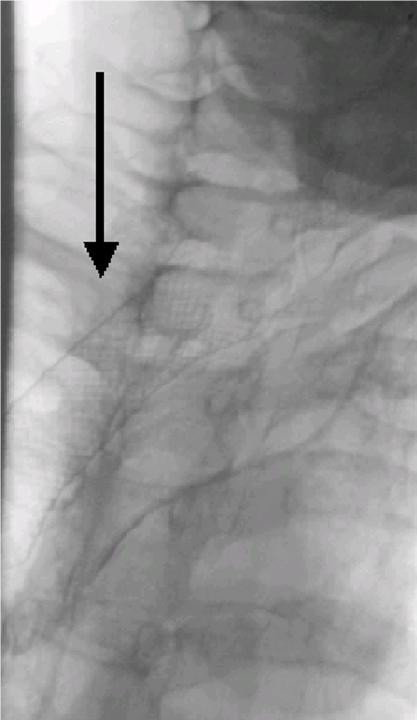
Nitinol self expanding stent placed within the esophageal cavity (arrow).

All endoscopic attempts to pass the obstruction failed, because the guide wire only entered the associated esophago-tracheal fistula. Therefore a retrograde endoscopic approach was undertaken. According to the previous esophageal resection with interposition of a gastric tube, radiologically guided percutaneous gastrostomy techniques [5] had to be rejected. Retrograde access to the esophageal lumen was obtained by open surgery and a duodenotomy (Figure 3). Through an endoscope a guide wire (Terumo, RF-GA35403M Standard, 0.035 inch) was pushed up and the esophageal obstruction was traversed, which simultaneously was monitored by a transnasal endoscope. Using a guiding catheter the esophageal stenosis was dilated and a naso-jejunal triluminal feeding tube was placed into the first jejunal loop. Subsequently the longitudinal duodenal incision was closed in a transverse fashion. Before closure of the abdominal wall a jejunostomy catheter was implanted to ensure sufficient enteral nutrition. 72 hours later, in a second step further endoscopic guided dilatation of the esophageal stenosis was repeated twice. Using a stiff wire (0.035 inch) placed under fluoroscopic control subsequent guide wired dilatation, up to 12.8 mm according to the method of Savary, was performed. In a third step a nitinol self-expanding fully covered stent, the so called Choo stent (M.I. Tech/MTW), was placed across the fistula under radiological and endoscopic control (Figure 4). After successful placement of the stent the upper end was located directly proximal from the stenosis at about 20 cm from frontal incisors and completely traversed the whole stenosis. As a result the patient felt neither foreign body sensation nor pain. A follow up contrast study, performed on the 4^th ^day after stent placement, showed the stent to be almost completely expanded without any signs of persisting leakage. Thereafter the patient was allowed to swallow liquid food, although only a small volume could be swallowed at a time. A few days later swallowing of semi solids and hypercaloric liquid food was possible and the patient was discharged.

Follow-up analyses revealed that the patient died 158 days after our treatment due to severe pleural effusion and diffuse pulmonary metastasis.

## Discussion

In patients with malignant esophageal obstruction and esophago-respiratory fistulae oral intake is limited by paroxysmal coughing, leading to profound malnutrition and death from recurrent pulmonary infections. Closure of the esophago-respiratory fistulae is the predominant goal of palliative therapy in this situation. Endoscopic placement of a covered expandable metallic stent is a well established minimal invasive approach [6]. In most cases stent placement begins with a transoral passage of a guide wire through the esophageal stenosis. Sophisticated utilization of angiographic techniques with catheters and guide wires enables dilatation even of high grade esophageal stenoses.

However in cases with complete obstruction and associated fistulae stenoses often remain impassable, because the guide wire constantly enters the wrong lumen of the fistula. Recently a new technique with retrograde passage of the stenotic segment has been described [7,8] and successfully applied in several centers [4,9-11]. In all cases a percutaneous gastric puncture was performed and an endoscope was directed into the distal esophagus enabling retrograde dilatation. Unfortunately this technique does not apply to those patients who initially underwent esophageal resection and subsequent interposition of a gastric tube. In our view postoperative adhesions and adjacent loops as well as the location of the small residual stomach clearly impede percutaneous punction in these patients. Even radiologically guided techniques for percutaneous punction are extremely difficult and pose a high-risk of perforation. Therefore we performed open surgery, identified the duodenum and entered an endoscope through a spare longitudinal incision. Via the gastric tube a guide wire was pushed up and the esophageal obstruction was traversed for subsequent stent placement.

In patients with esophageal carcinoma local recurrence as well as ERF are frequently observed, despite of radical surgery and adjuvant radio-chemotherapy. If transoral passage and stent placement is not possible, these patients urgently need alternative approaches for successful palliation. Due to the interposition of a gastric tube, postoperative anatomy is complex and retrograde endoscopy via a percutaneous gastrostomy has not been described in the current literature.

## Conclusion

In our view open surgery is a safe means to access the gastric tube via a duodenal incision, enabling retrograde endoscopic dilatation of the obstructed segment as well as simultaneous implantation of a jejunostomy catheter for sufficient enteral nutrition. This approach should be considered for high grade esophageal obstruction and ERF, when antegrade passage of the lumen is not possible. Surgery is warranted even if retrograde esophageal passage might fail, because open implantation of a jejunostomy catheter for enteral nutrition remains the only and ultimate palliative option in this situation.

## Competing interests

The author(s) declare that they have no competing interests.

## Authors' contributions

**RA**: Reviewed the current literature, drafted the manuscript and made substantial intellectual contributions to the article; **CK**:Initiated the publication of this case, helped to draft the manuscript and revised it critically; **SM**:Performed the endoscopic procedures, helped to draft the manuscript and revised it critically. **KP**: Performed the X-ray examinations and supplied digital artwork. **RMA**: Performed endoscopic procedures and helped in drafting the manuscript; **IÖ**: Participated in the design of this article and coordinated and helped to draft the manuscript. **KWT: **Performed the surgical treatment of the patients, helped to draft the manuscript and revised it critically.

All authors read and approved the manuscript.
